# Selumetinib as an Effective Therapy of Histiocytic Sarcoma Evolving From a B‐Cell Acute Lymphoblastic Leukaemia

**DOI:** 10.1002/jha2.70216

**Published:** 2026-03-05

**Authors:** Laetitia Largeaud, Charlotte Syrykh, Julie Vial, Alban Canali, Isabelle Luquet, François Vergez, Stéphanie Dufrechou, Naïs Prade, André Baruchel, Bastien Gerby, Marie Nolla, Eric Delabesse, Marlène Pasquet

**Affiliations:** ^1^ Laboratory of Hematology Institut Universitaire du Cancer de Toulouse, CHU Toulouse Toulouse France; ^2^ Université De Toulouse, Inserm, Centre National de la Recherche Scientifique, Université Toulouse III‐Paul Sabatier Centre de Recherches en Cancérologie de Toulouse Toulouse France; ^3^ Equipe Labellisée Ligue Contre le Cancer 2023 Toulouse France; ^4^ Equipe Labellisée Institut Carnot Opale Toulouse France; ^5^ Laboratory of Anatomo‐Pathology Institut Universitaire du Cancer de Toulouse, CHU Toulouse Toulouse France; ^6^ Department of Pediatric Radiology CHU Toulouse Toulouse France; ^7^ Department of Pediatric Hematology and Immunology Hôpital Robert Débré APHP Université Paris Cité Paris France; ^8^ Department of Pediatric Hematology and Immunology CHU Toulouse Toulouse France

**Keywords:** B cell acute lymphoblastic leukemia, histiocytic sarcoma, transdifferentiation, MEK inhibitor

## Abstract

**Introduction:**

Histiocytic sarcoma (HS) is a rare neoplasm derived from non‐Langerhans histiocytic cells, exceptionally arising from B‐ALL.

**Methods:**

We present the case of a child with high‐risk B‐ALL with PAX5 P80R mutation.

**Results:**

Despite initial remission, a chemoresistant paravertebral mass was identified as HS. A shared IGK/TCRB rearrangements and PAX5 alterations between the leukaemic and histiocytic clones suggested transdifferentiation driven by PAX5. A somatic MAP2K1 mutation in the HS component prompted selumetinib treatment, leading to a rapid response.

**Conclusion:**

This case underscores the role of PAX5 in lineage plasticity and highlights the potential of targeted MEK inhibition in MAPK‐driven HS arising from B‐ALL.

**Trial Registration:**

The authors have confirmed clinical trial registration is not needed for this submission

## 1 Introduction

Histiocytic sarcoma (HS) is characterized by tissue infiltration of malignant cells with morphological and phenotypic features of macrophages. Such histiocytic/dendritic cell neoplasms have rarely been reported in an acute lymphoblastic leukaemia (ALL), lacking a standard treatment due to their rarity. Its prognosis remains poor in the few case reports published [1]. We report here a case of PAX5‐mutated and *MAP2K1*‐mutated HS arising at the diagnosis of B‐cell ALL who responded to selumetinib, a MEK1/2 inhibitor, allowing a bridge treatment to bone marrow transplant and cure.

## 2 Materials and Methods

**FIGURE 1:**
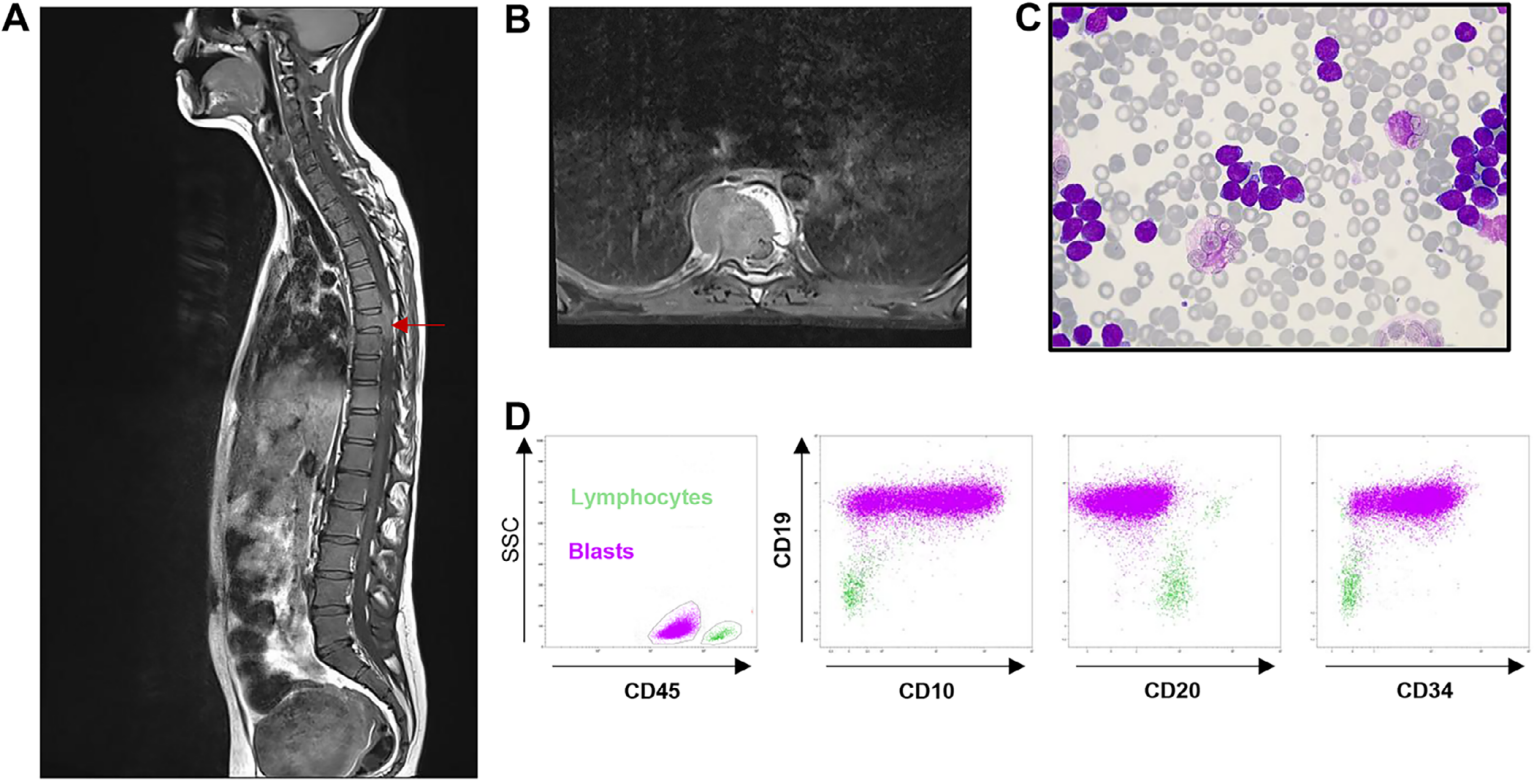
B‐ALL diagnosis with extramedullary lesions. (A, B) Sagittal T1 and axial T1 FS gadolinium MRI sequences showing right paravertebral mass of T7 (34 × 29 mm) with vertebral compression of T7, lytic lesion of the upper plateau of L1, diffuse medullary infiltration in hyposignal T1 of the various vertebral bodies. (C) (MGG, ×63) Lymphoblastic cells with high nuclear/cytoplasmic ratio, basophilic cytoplasm and immature chromatin. (D) Flow cytometry analysis of leukaemic cells and lymphocytes showing expression of CD10, CD19, CD20 and CD34 markers on the cell surface.

A 13‐year‐old girl was referred to our unit because of asthenia and chronic back pain. A computed tomography (CT) and magnetic resonance imaging (MRI) scan revealed a large lytic lesion infiltrating the right side of the T7 vertebral body with pathological compression and epiduritis from T6 to T8 levels (Figure 1A,B). Cerebrospinal fluid showed no abnormality. Bone marrow aspiration and flow cytometry were performed and showed 96% lymphoblasts (Figure 1C) whose phenotype led to the diagnosis of CD10+ CD19+ CD20‐ B‐ALL (Figure 1D). The leukaemic cells were associated with IGH, IGK and TCRB clonal rearrangements, a dicentric (9;20)(p12;q11) chromosome resulting in 9p (deletions of *PAX5*, *JAK2*, *CDKN2A*/*B* genes) and 20q deletion, and presence of *PAX5* (p.Pro80Arg), *KMT2 D* (p.Phe301Tyr) and *PTPN11* (p.Asn200Tyr) mutations along with a subclonal *NRAS* mutation (p.Gly13Asp).

## 3 Results

**FIGURE 2:**
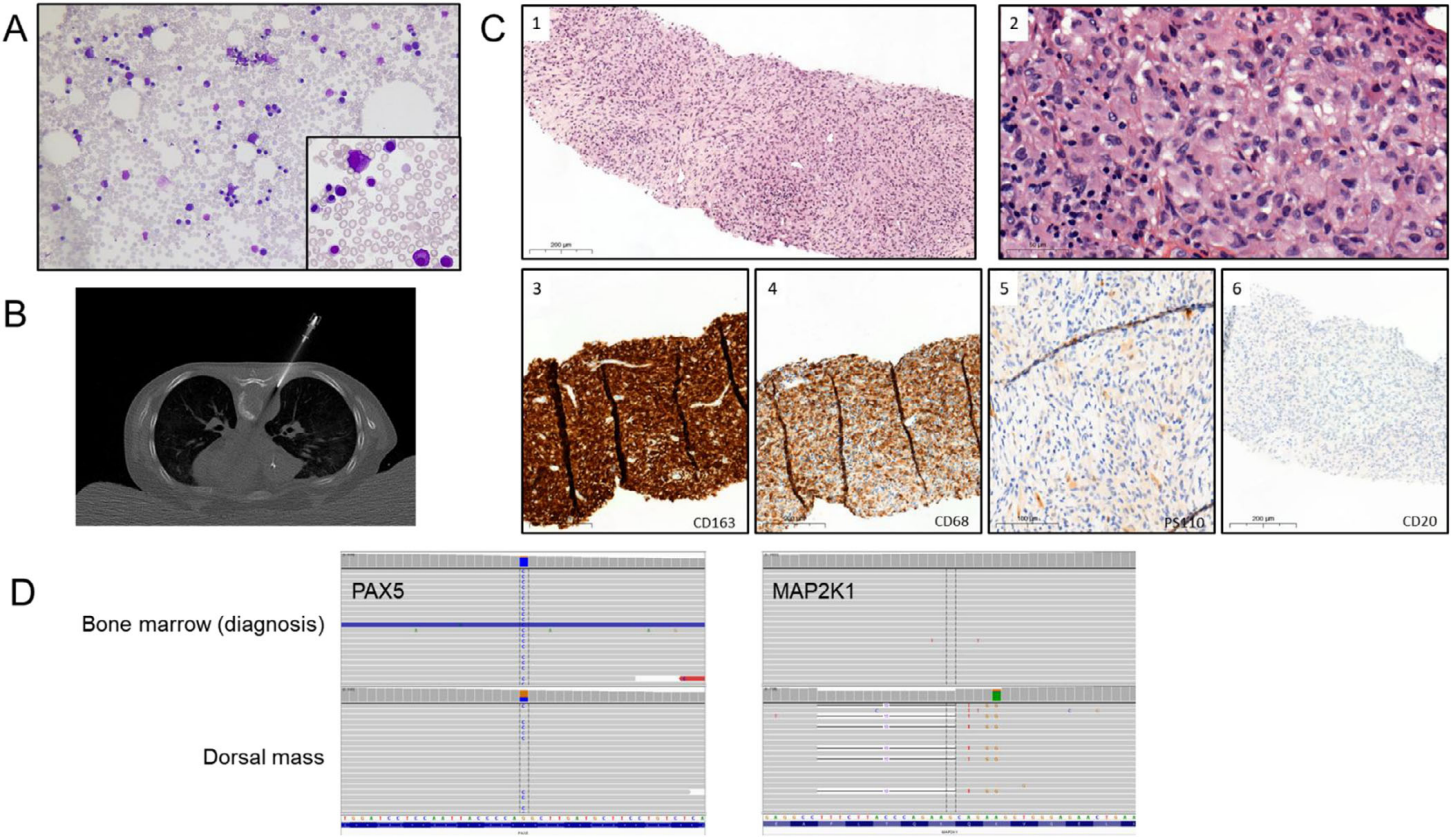
Response dissociation with progression of extramedullary lesion. (A) (MGG, ×10 and ×63) Bone marrow evaluation 1 month after the beginning of induction therapy. Decrease of granulocytic cells associated with absence of excess of blast cells. (B) CT biopsy of T7 dorsal mass in axial section. (C) (1). (H&E, ×10) and (2). (H&E, ×40) Diffuse sheets of tumoral cells with abundant eosinophilic cytoplasm, indistinct cellular borders and oval or grooved nuclei. (3). (CD163, ×10) and (4). (CD68 PGM1, ×10) Tumoral cells expressed the histiocytic markers CD163 and CD68. (5). (PS110, ×20) and (6). (CD20, ×10). The infiltrate was negative for PS100 and CD20. (D) Detection of *PAX5* P80R mutation in bone marrow samples at diagnosis and in dorsal mass. *MAP2K1* mutation detected only in dorsal mass.

**FIGURE 3:**
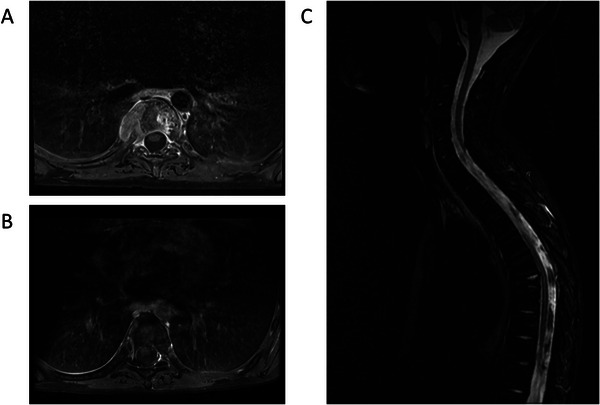
Selumetinib response pre‐HSCT transplant and persistence of long‐term remission. (A) Axial T1FS gadolinium MRI sequences showing reduction of the extra‐osseous component. (B) No residual paravertebral mass at T7 visualized in axial T1FS gadolinium MRI sequence. (C) Regression of the sagittal STIR hypersignal of the T7 vertebra with no sign of epiduritis or spinal cord abnormalities but persistent vertebral compression at T7.

The child was treated with a polychemotherapy induction treatment dedicated to high‐risk B‐cell ALL according to standard national CALL‐F01 protocol (ClinicalTrials.gov Identifier: NCT02716233) with four drugs (prednisone, vincristine, asparaginase and doxorubicin), with a reinforced intrathecal therapy due to the central nervous system involvement (epiduritis). Post induction evaluation showed complete remission with an absence of blasts in bone marrow evaluation (Figure 2A) associated with a minimal residual disease (MRD) at 1‐month post induction at 10^−4^. However, the patient had persistent back pain requiring morphinics and the MRI showed a continued settling of the T7 vertebra with an increase of the tissue lesion, measuring 40 mm in width × 35 mm in thickness × 34 mm in height, as opposed to the previous 33 × 32 × 30 mm, suggesting that the lesion was strongly resistant to chemotherapy. The spinal cord was not affected. A paravertebral mass biopsy (Figure 2B) was performed during consolidation chemotherapy revealing a diffuse proliferation of atypical tumoral cells with histiocytic appearance (Figure 2C). These tumoral cells expressed the histiocytic markers CD163 and CD68 without Langerhans cell markers (PS100, CD1a and Langerin being negative). CD3, CD20, CD21, CD23, CD34, CD117 and TdT markers were negative. Molecular analyses found the same IGK and TCRB rearrangements, *PAX5* p.Pro80Arg mutation (VAF: 86%) and *PAX5* and *CDKN2A*/B deletions identified at diagnosis, suggesting a common clonal origin of the B‐ALL and the HS. An additional *MAP2K1* (i.e., *MEK1*) mutation (p.Phe53_Lys59delinsLeuGlyl, VAF: 17%) was detected related to histiocytic morphology (Figure 2D). In contrast, *KMT2 D* (VAF: 48%), *PTPN11* (VAF: 33%) and *NRAS* (VAF: 3%) mutations identified at diagnosis were not detected. After completion of consolidation treatment, four high dose methotrexate courses were administrated due to the initial CNS involvement. Evaluation revealed persistence of bone marrow remission with negative MRD, without modification of the back lesion. At this time, considering the presence of the specific *MAP2K1* mutation in the HS, we introduced the oral selective MEK inhibitor selumetinib associated with dexamethasone pulses according to dose recommended in the SeluDex trial opened in 2018 (ClinicalTrials.gov Identifier: NCT03705507). One month after selumetinib initiation, the dorsal pain disappeared along with a significant reduction of the extra‐osseous component and reduction of the hyperintense T2 signal (Figure 3A). Selumetinib treatment was maintained with no side effects and persistence of haematological remission in the bone marrow until bone marrow transplantation. After a conditioning regimen with a total body irradiation, Endoxan and Fludarabin, the patient was allografted with an umbilical cord blood and is still in complete haematologic remission 2 years after transplantation. The vertebra lesion progressively regressed at 12 months and disappeared at 18 months with no significant vertebral or paravertebral contrast uptake and regression of the STIR hypersignal of the T7 vertebra and no sign of epiduritis or spinal cord abnormalities (Figure 3B,C).

## 4 Discussion

Very few cases of histiocytic neoplasms are associated with ALL. The case presented here illustrates the efficacy of targeted molecular therapies in these rare diseases with few therapeutic options, the Phase I/II SeluDex clinical trial being interrupted due to a lack of recruitment [2]. An initial series of 15 patients confirmed the ‘atypical histological presentation’ ranging from Langerhans' cell histiocytosis to HS [3]. Identification of cell of origin shared with the leukaemic clone was only achieved in 7 out of 15 patients, confirming the need for an accurate histopathology diagnosis and advanced molecular analyses in these cases.

Given the complete haematological remission in the bone marrow and the persistence of active disease in the vertebra, we switched to MEK‐targeted therapy treatment to expect a good response before the allograft. This treatment allowed a rapid and significant improvement without side effects. Most importantly, chronic pain resolved rapidly, and the patient could have normal activities and restarted adapted physical activities. In a recent study, selumetinib‐dexamethasone combination showed a synergistic efficacy in vitro and in an orthotopic mouse model engrafted with RAS pathway‐activated primary‐derived ALL cells [4].

Regarding prognosis, a recent review of 30 patients confirmed the bad prognosis of this entity with 19 deaths, of whom 12 related to histiocytic/dendritic cell neoplasms [1]. Twenty‐one patients never achieved remission of the histiocytic/dendritic cell disorder, arguing for early control of the disease and novel therapies such as the one used here. Interestingly, Fenu et al. previously reported a case of B‐ALL transdifferentiation into HS following CAR‐T cell therapy, a treatment now widely used for B‐ALL. Such case series highlight the importance of raising clinicians' awareness about these post‐treatment complications and emphasize that molecular characterization is crucial in this context, given the successful response to MEK inhibitor therapy observed in our case [5].

Finally, there is still a debate whether histiocytic/dendritic cell neoplasms occur in the context of ALL as trans‐differentiation, dedifferentiation or if a common progenitor cell with ALL is involved [3, 6]. *PAX5* encodes a paired box domain (PBD) transcription factor considered as the guardian of B‐cell identity. Indeed, PAX5 controls the irreversible commitment of lymphoid progenitors to the B‐cell lineage by activating the transcription of B‐cell‐specific programs and by suppressing alternative lineage choices [7, 8, 9]. Its homozygous deletion in the mouse leads to a blockade of differentiation at the transition between the pre‐pro‐B and pro‐B stages [10]. At the functional level, uncommitted Pax5^−/−^ B‐cells are able to differentiate into several hematopoietic cell types [7, 11, 12, 13]. Finally, it has been shown that Pax5 inactivation in mature B‐cells leads to the reactivation of lineage‐inappropriate genes, allowing for their conversion into functional T cells by transdifferentiation (or dedifferentiation) to early uncommitted progenitors [14]. According to the critical role of Pax5 in the B‐cell development and the degree of plasticity of B‐cells, we hypothesize that the detection of the hypomorphic *PAX5 P80R* variant associated with the deletion of the second *PAX5* allele have led to an important loss‐of‐function of PAX5, sufficient to induce transdifferentiation of blast cells into the histiocytic lineage. The detection of similar molecular abnormalities and particularly identical IGK and TCRB‐rearrangements in both neoplasms also supports the concept of lineage plasticity, suggesting a transdifferentiation of B‐ALL into HS. Moreover, it has been shown that the *PAX5 P80R* mutation defines an independent and exclusive B‐ALL subtype in patients [15, 16], supporting the notion that *PAX5 P80R* mutation is a primary oncogenic event in the multi‐step process of leukaemia. This could explain why the common clonal origin of B‐ALL and the HS is characterized by the presence of the *PAX5 P80R* mutation.

## 5 Conclusion

The case described here provides a proof of concept that demonstrated the efficacy and safety of the use of selumetinib in the context of *MEK*‐mutated histiocytic neoplasm and ALL, which could be used as a bridge to transplant to ensure a long‐term control of this rare and aggressive disease.

## Author Contributions

L.L., C.S., I.L., N.P., S.D., M.N., B.G., E.D., M.P. analysed the data. L.L. and M.P. wrote the manuscript. F.V., A.C. performed the flow cytometry experiment and analysed the data. C.S. was responsible for the histology review. J.V. provided the imaging. E.D. revised the manuscript. All authors have read and approved the final submitted version of the manuscript.

## Funding

This study was supported by institutional grants from INSERM, the Ligue Contre le Cancer and the associations Laurette Fugain, 111 des arts, Enfants Cancers et Santé, and Constance la petite guerrière astronaute.

## Ethics Statement

Written informed consent was obtained from patient and parents according to local regulations.

## Conflicts of Interest

The authors declare no conflicts of interest.

## Data Availability

The data that support the findings of this study are available from the corresponding author upon reasonable request.

## References

[1] G. Hubert , H. Bittencourt , C. Laverdière , et al., “Clinical Response to Dabrafenib and Chemotherapy in Clonally‐Related Histiocytosis and Acute Lymphoblastic Leukemia,” Haematologica 108, no. 6 (2023): 1707–1712, 10.3324/haematol.2022.281926. 36384252 PMC10230417

[2] T. Menne , D. Slade , J. Savage , et al., “Selumetinib in Combination With Dexamethasone for the Treatment of Relapsed/Refractory RAS‐Pathway Mutated Paediatric and Adult Acute Lymphoblastic Leukaemia (SeluDex): Study Protocol for an International, Parallel‐Group, Dose‐Finding With Expansion Phase I/II Trial,” BMJ Open 12, no. 3 (2022): e059872. 10.1136/bmjopen-2021-059872 PMC8900053 35246426

[3] E. C. C. Castro , C. Blazquez , J. Boyd , et al., “Clinicopathologic Features of Histiocytic Lesions Following ALL, With a Review of the Literature,” Pediatric and Developmental Pathology 13, no. 3 (2010): 225–237, 10.2350/09-03-0622-OA.1. 19642834

[4] E. C. Matheson , H. Thomas , M. Case , et al., “Glucocorticoids and Selumetinib Are Highly Synergistic in RAS Pathway‐Mutated Childhood Acute Lymphoblastic Leukemia Through Upregulation of BIM,” Haematologica 104, no. 9 (2019): 1804–1811, 10.3324/haematol.2017.185975. 30655370 PMC6717586

[5] E. M. Fenu , E. Margolskee , and V. Pillai , “Transdifferentiation of B‐Lymphoblastic Leukemia to Histiocytic Sarcoma After Immunotherapy,” American Journal of Hematology 98, no. 8 (2023): E216–E218, 10.1002/ajh.26983. 37259821

[6] M. Bleeke , P. Johann , S. Gröbner , et al., “Genome‐Wide Analysis of Acute Leukemia and Clonally Related Histiocytic Sarcoma in a Series of Three Pediatric Patients,” Pediatric Blood & Cancer 67, no. 2 (2020): e28074, 10.1002/pbc.28074. 31737984

[7] S. L. Nutt , B. Heavey , A. G. Rolink , and M. Busslinger , “Commitment to the B‐Lymphoid Lineage Depends on the Transcription Factor Pax5,” Nature 401, no. 6753 (1999): 556–562, 10.1038/44076. 10524622

[8] A. Souabni , C. Cobaleda , M. Schebesta , and M. Busslinger , “Pax5 Promotes B Lymphopoiesis and Blocks T Cell Development by Repressing Notch1,” Immunity 17, no. 6 (2002): 781–793, 10.1016/S1074-7613(02)00472-7. 12479824

[9] K.‐P. Nera , P. Kohonen , E. Narvi , et al., “Loss of Pax5 Promotes Plasma Cell Differentiation,” Immunity 24, no. 3 (2006): 283–293, 10.1016/j.immuni.2006.02.003. 16546097

[10] P. Urbanek , “Complete Block of Early B Cell Differentiation and Altered Patterning of the Posterior Midbrain in Mice Lacking Pax5/BSAP,” Cell 79, no. 5 (1994): 901–912, 10.1016/0092-8674(94)90079-5. 8001127

[11] A. G. Rolink , S. L. Nutt , F. Melchers , and M. Busslinger , “Long‐Term In Vivo Reconstitution of T‐Cell Development by Pax5‐Deficient B‐Cell Progenitors,” Nature 401, no. 6753 (1999): 603–606, 10.1038/44164. 10524629

[12] C. Schaniel , L. Bruno , F. Melchers , and A. G. Rolink , “Multiple Hematopoietic Cell Lineages Develop In Vivo From Transplanted Pax5‐Deficient Pre‐B I–Cell Clones,” Blood 99, no. 2 (2002): 472–478, 10.1182/blood.V99.2.472. 11781227

[13] S. Hoflinger , K. Kesavan , M. Fuxa , et al., “Analysis of Notch1 Function by In Vitro T Cell Differentiation of Pax5 Mutant Lymphoid Progenitors,” Journal of Immunology 173, no. 6 (2004): 3935–3944, 10.4049/jimmunol.173.6.3935. 15356142

[14] C. Cobaleda , A. Schebesta , A. Delogu , and M. Busslinger , “Pax5: The Guardian of B Cell Identity and Function,” Nature Immunology 8, no. 5 (2007): 463–470, 10.1038/ni1454. 17440452

[15] Z. Gu , M. L. Churchman , K. G. Roberts , et al., “PAX5‐Driven Subtypes of B‐Progenitor Acute Lymphoblastic Leukemia,” Nature Genetics 51, no. 2 (2019): 296–307, 10.1038/s41588-018-0315-5. 30643249 PMC6525306

[16] M. Passet , N. Boissel , F. Sigaux , et al., “PAX5 P80R Mutation Identifies a Novel Subtype of B‐Cell Precursor Acute Lymphoblastic Leukemia With Favorable Outcome,” Blood 133, no. 3 (2019): 280–284, 10.1182/blood-2018-10-882142. 30510083

